# Comparison of effectiveness of skin antiseptics using conventional application with forceps and gauze swabs, single-use applicator, or by wetting skin with a low or high density of sebaceous glands

**DOI:** 10.3205/dgkh000596

**Published:** 2025-11-21

**Authors:** Torsten Koburger-Jannsen, Axel Kramer

**Affiliations:** 1 Hygiene North GmbH, Greifswald, Germany; 2 2Institute of Hygiene and Environmental Medicine, University Medicine Greifswald, Germany

**Keywords:** preoperative skin antisepsis, efficacy of single-use applicator, efficacy of forceps with gauze swab, efficacy of wetting; propan-2-ol, combination chlorhexidine digluconate/propan-2-ol, sustainability of single-use applicator, sustainability of conventional application

## Abstract

**Background:**

The preoperative application of skin antiseptics can be performed conventionally with sterile forceps and a sterile gauze swab or with a ready-made single-use applicator. Since the latter is more ecologically than the conventional procedure, an investigation into whether the application method influences the antiseptic efficacy is warranted.

**Method:**

The comparison was performed on the upper arm (low density of sebaceous glands) and forehead (high density of sebaceous glands) of volunteers according to test method 13 of the Association for Applied Hygiene for the certification of skin antiseptics in Germany. The antiseptic, 70% v/v propan-2-ol +2% w/v chlorhexidine digluconate (P/CHG), was applied either with the applicator, with forceps, or by gentle wetting without rub-in. After rub-in or wetting, samples were taken from the application area with sterile swabs, transferred into tryptic soy broth and plated onto tryptic soy agar. The reduction of the skin flora was calculated based on the number of colony forming units before and after antisepsis application.

Additionally, the formulation P/CHG was compared with the reference standard 70% v/v propan-2-ol (P) for testing skin antiseptics in Germany.

**Results:**

There was no difference in antiseptic efficacy between the use of applicator or forceps. However, if the skin was only gentle wetted with the antiseptic, the efficacy was significantly lower than after rub-in with the applicator or forceps.

In one trial, the antiseptic P/CHG tended to be more effective or, in another trial, was statistically significantly more effective than P when tested on skin with few sebaceous glands.

**Discussion:**

The results underline the necessity of thoroughly rubbing-in the antiseptic for preoperative skin antisepsis instead of merely wetting the skin.

The literature confirms the higher efficacy of the P/CHG antiseptic compared to P (the reference for testing skin antiseptics in Germany), based on the criterion of rate of surgical site infections (SSI).

**Conclusion:**

According to the GRADE (Grading of Recommendations Assessment, Development and Evaluation)-based classification of evidence, the results presented here are of “high evidence”. Thus, for preoperative skin antisepsis, P/CHG is to be used instead of alcohol-based formulations lacking CHG, due to the sustained efficacy of CHG.

As the efficacy of applying the antiseptic with forceps and gauze swab or with single-use applicator does not differ on skin with few or many sebaceous glands, the single-use applicator should be preferred due to the guaranteed standardization of application, the time savings compared to the conventional method, and more favorable economic and ecological sustainability.

## Zusammenfassung

**Hintergrund:**

Die präoperative Applikation des Hautantiseptikums kann konventionell mit steriler Kornzange und sterilem Tupfer oder mit einem fertig konfektionierten Einmalapplikator durchgeführt werden. Weil ersterer im Ergebnis des Lifecycle Assessments nachhaltiger als das konventionelle Vorgehen ist, sollte untersucht werden, ob die Applikationsart einen Einfluss auf die antiseptische Wirksamkeit hat.

**Methode:**

Der Vergleich wurde am Oberarm (talgdrüsenarme Haut) und auf der Stirn (talgdrüsenreiche Haut) von Probanden gemäß der Prüfmethode 13 des Verbunds für Angewandte Hygiene zur Zertifizierung von Hautantiseptika in Deutschland durchgeführt. Das Antiseptikum, 70% v/v Propan-2-ol (P) +2% w/v Chlorhexidindigluconat (CHG), wurde mittels Applikator, mittels Kornzange und Tupfer oder durch Benetzung ohne mechanisches Einreiben auf die Testfläche aufgetragen. Nach unterschiedlichen Expositionszeiten wurden mit sterilen Tupfern Proben vom Applikationsareal entnommen, in Trypsin-Soja-Bouillon überführt und auf Trypsin-Soja-Agar ausplattiert. Die Reduktion der Hautflora wurde anhand der Anzahl Kolonie bildender Einheiten vor und nach der Antisepsis berechnet.

Zusätzlich wurde die Formulierung P/CHG mit dem Referenzstandard P zur Prüfung von Hautantiseptika in Deutschland verglichen.

**Ergebnisse:**

Zwischen der Anwendung des Applikators oder der Kornzange unterschied sich die antiseptische Wirksamkeit nicht. Wurde die Haut mit dem Antiseptikum lediglich benetzt, war die Wirksamkeit jedoch signifikant geringer als nach Einreiben mittels Applikators oder Kornzange.

Das Antiseptikum P/CHG, getestet auf talgdrüsenarmer Haut, war in einem Versuch tendentiell, in einem weiteren Versuch signifikant wirksamer als P.

**Diskussion:**

Die Ergebnisse unterstreichen die Notwendigkeit, das Antiseptikum zur präoperativen Hautantiseptik anstelle der Benetzung der Haut gründlich einzureiben. Dabei ist es ohne Einfluss, ob die Einreibung mittel Applikator oder Kornzange erfolgt.

Die höhere Wirksamkeit des Antiseptikums P/CHG im Vergleich zu P, der Referenz zur Prüfung von Hautantiseptika, wurde in der Literatur am Endpunkt der SSI-Rate bestätigt.

**Schlussfolgerung:**

Zur präoperativen Hautantiseptik ist das Antiseptikum P/CHG auf Grund der remanenten Wirkung von CHG anstelle alkoholischer Formulierungen ohne Zusatz von CHG mit hoher Evidenz anzuwenden.

Da sich die Wirksamkeit des Auftragens des Antiseptikums mittels Einwegapplikator oder mittels Kornzange und Tupfer sowohl auf talgdrüsenarmer als auch auf talgdrüsenreicher Haut nicht unterscheidet, ist dem Einwegapplikator wegen der gewährleisteten Standardisierung der Auftragung, der Zeitersparnis im Vergleich zum konventionellen Vorgehen und der günstigeren Nachhaltigkeit der Vorzug zu geben.

## Introduction

The introduction of the ready-made single-use applicator for preoperative application of skin antiseptic was developed with the aim to standardize application and thus possibly achieve constant or better efficacy of skin antisepsis. Another advantage of the applicator is the more favorable lifecycle assessment (LCA) compared to application with forceps and gauze swabs [1]. As a basis for choosing the technique of application, this study investigated whether there is a difference in antiseptic efficacy between application with the disposable applicator, conventional application, or only gentle wetting.

## Methods

### Ethics statement 

The study was conducted in compliance with the WMA Declaration of Helsinki, Ethical Principles for Medical Research [2], the State Data Protection Act and the General Data Protection Regulation as well as the Professional Code of Conduct for Physicians in Mecklenburg Western Pomerania [3]. In addition, the statement from the Federal Institute for Drugs and Medical Devices of Germany (BfArM) [4] affirms “that testing of chemical disinfectants and antiseptics phase 2, step 2, according to the European Norm EN 12791, are explicitly not treated by the BfArM as clinical trials within the meaning of the Medicinal Products Act”. This means that the requirements for pharmacological monitoring required for clinical trials and the fulfillment of other requirements in accordance with good clinical practice are not necessary.”

### Study design 

According to the test method 13 of the Association of Applied Hygiene (VAH) for the certification of skin antiseptics in Germany [5], the test was performed on the upper arm – which has a low density of sebaceous glands – and on the forehead, which has a high density of sebaceous glands. The exclusion criteria were:

- skin had been treated with disinfectants or antiseptics within three days prior to the test,
- antibiotic therapy had been administered within three days prior to the test (possibly altered skin flora),
- dermatosis in the test area,
- fever.

To participate in the study, the volunteers were required to sign an informed consent.

In 6 trials (Table 1 (Tab. 1)), the efficacy of formulations based on 70% v/v propan-2-ol (P) +2% w/v chlorhexidine digluconate (CHG) from different manufacturers was compared when applied with single-use applicator, forceps and gauze swab, or only by gently wetting the skin. In 2 trials, P was included in the comparison. Depending on the antiseptic, different neutralizers were used. The effectiveness of the neutralization was tested according to method 9 of the VAH [5]. 

### Determination of pre-values 

The pre-values were determined by swabbing a separate test field (2x2.5 cm) on the other upper arm that had not come into contact with the antiseptic. Using the cotton swab moistened in 5 ml tryptic soy broth (TSB, Carl Roth GmbH & Co. KG Karlsruhe, Germany) with neutralizing agent, which is necessary to neutralize the antiseptic after skin antisepsis, the marked test field was thoroughly swabbed for 15 seconds. Care was taken to ensure that the swab did not go beyond the edges. The swabs were then transferred to 5 ml TSB/neutralizer and shaken for 30 s at high frequency in a test-tube shaker. This collection liquid was further diluted to 1:10 in TSB/neutralizer. 0.1 ml each of undiluted and diluted sample were plated onto tryptic soy agar plates (TSA, Carl Roth GmbH + Co. KG Karlsruhe, Germany) and cultivated for 48 h at 36±1°C. 

### Testing on the upper arm 

The skin antiseptic was applied on the marked test field on the left arm with the 3 ml single-use applicator or on the right arm with forceps and sterile gauze swabs, size 6 (Schlinggazetupfer Fuhrmann GmbH, Much, Germany) to simulate surgical skin antisepsis as realistically as possible. Regarding the comparative nature of the study, the exposure times were set between 15 seconds and 2 min only. 

The applicator was used as follows. After breaking the ampoule inside the test product, it was ensured that the sponge pad of the applicator became completely soaked with the antiseptic by pressing it flat into the blister pack from which it was taken. The previously marked area on the right arm of about 4x12 cm was well moistened by repeatedly stroking the applicator up and down for 15s under constant moderate pressure that neither damaged the skin nor was perceived as unpleasant by the test subjects skin that was clearly noticeable yet not perceived as stressful for the volunteers nor damaging the skin. Immediately after the exposure time, samples were taken from a 2.5x2 cm test area within the upper center of the application area using the standard swab technique according to the VAH. If two exposure times were tested, the second sample was taken from the lower center of the application area. Thus, the application time was included in the contact time. For each volunteer, a new applicator was used.

The forceps were used as follows. The gauze swabs, applied with the forceps, were soaked with 5 ml of the antiseptic in a Petri dish. Immediately thereafter, the previously marked application area of about 4x12 cm was well moistened by repeatedly stroking the gauze ball up and down for 15 s under constant moderate pressure that neither damaged the skin nor was perceived as unpleasant by the test subjects. Immediately post-exposure, samples were taken from a 2.5x2 cm test area within the upper center of the application area using the standard VAH swab technique. If two exposure times were tested, the second sample was taken from the lower center of the application area. Thus, the application time was included in the contact time. A new applicator was used for each volunteer.

To wet the skin, sterile, well-moistened gauze swabs were briefly pressed on the skin without rub-in.

Post-values were taken right at the end of the exposure from a standard sized application area (12.5 cmx4 cm) using the VAH standard swab technique. The application area was not washed or treated with skin cream until the post-value was determined. 

### Testing on the forehead 

The applicator was used as above. After breaking the ampoule, it was ensured that the applicator’s sponge pad became completely soaked (3–4 s) by circling 3 times in a sterile Petri dish with light pressure. The test area (2.5 cmx2 cm) was then wetted by repeatedly stroking the applicator back and forth for 30 s under constant moderate pressure that neither damaged the skin nor was perceived as unpleasant by the test subjects. For each volunteer, a new applicator was used. 

Pre-values were determined by swabbing a separate test area that had not been exposed to the antiseptic. Post-values were taken right at the end of the exposure from a standard sized (2.5 cmx2 cm) sampling area in the middle of the 12.5 cm^2^ test area using the standard VAH swab technique.

For application with forceps, the gauze swab was placed in a sterile 9-cm Petri dish and soaked with 4.2 ml for 3 s by distributing it evenly on the gauze ball using a pipette. The 4.2-ml volume had been identified as ideal for avoiding excess spilling of liquid during the application. For rub-in, the gauze swab was gripped laterally in the middle by the dressing forceps and used for repeated application of the antiseptic on a total area of 2.5x2 cm for 30 s. 

Post-values were taken right at the end of the exposure from a standard sized application area (1.25 cmx4 cm) using the VAH standard swab technique. The application area was not washed or treated with skin cream until the post-value had been determined. 

### Calculation of reduction 

CFU reduction calculation was performed according to DIN EN 12791 [6]. After counting the plates, the colony forming units per 1 ml were converted into decadic logarithms. The reduction (R) was calculated using the following formula: 

R=lg (pre-value)–lg (post-value)

For the statistical evaluation, the Wilcoxon signed-ranks test was used to compare the reduction of the antiseptics depending of the application technique or the antiseptic. Finally, the results were checked for consistency. Due to the predominantly exploratory nature of testing, the significance level was set at p=0.1. A one-sided test was performed in accordance with VAH method 13 requirements [5].

## Results

### Trial 1 

P/CHG antiseptic applied with forceps was significantly more effective than when applied with the applicator (Table 2 (Tab. 2)). For the calculated smaller sum of ranks (sum of positive ranks=42), the p-value was determined with p=0.008 (1-tailed). 

The influence of the application technique on skin was evaluated by a dermatologist who examined redness/flare, turgor/swelling, weeping, scaling, eczema and miscellaneous on a scale of 1 to 10 immediately before application, at the end of the 30-s contact time, 30 s and again 24 h after the test. Subjects were asked to evaluate the parameters itching, dysesthesia, stinging, pain and miscellaneous at the same time. A minor degree of redness/flare was observed in almost all subjects after the 30-s application; the scores of the 20 subjects added up to 33 using the applicator and 39 using the gauze/forceps. Five subjects reported minor stinging after the 30-s application; their scores added up to 2 for the applicator and 15 for the gauze/forceps application technique.

### Trial 2 

The two application techniques led to essentially identical results after 30 s of rubbing-in and post-treatment after 24 hours (Table 3 (Tab. 3)).

### Trial 3 

Without mechanical rub-in of the antiseptic, the efficacy was significantly lower (p=0.000, 1-tailed) than when using the applicator (Table 4 (Tab. 4)).

### Trial 4 

After application with forceps or applicator on the upper arm, the reduction did not differ either after 15 s or 30 s exposure time. 

In contrast, the P/CHG antiseptic applied with the forceps was significantly more effective than P/CHG applied with the applicator. The calculated smaller sum of ranks (sum of negative ranks=1.00) was statistically significantly smaller (p=0.025)(1-tailed) (Table 5 (Tab. 5)).

### Trial 5 

The efficacy of the formulations based on P/CHG did not significantly differ after application with the applicator or with the forceps on the upper arm (Table 6 (Tab. 6)).

Both formulations based on P/+CHG were only slightly, statistically not significantly more effective than the reference standard 70% v/v propan-2-ol.

### Trial 6 

The effectiveness of the antiseptic on the forehead also did not differ significantly between application by forceps or by applicator (Table 7 (Tab. 7)).

## Discussion

In the past, numerous guidelines have addressed the choice of antiseptics, but not the method of their application. However, the application technique determines the possibility of standardization, with its resulting safety and practicability [7]. In this study, it has been proven for the first time that the application of skin antiseptic without mechanical rub-in, i.e., only after gently wetting the skin, was, as expected, significantly less antiseptically effective than rub-in with the single-use applicator.

In contrast, different aspects have played a role in deciding between application with the single-use applicator or forceps. In trial 1, P/CHG applied with forceps was significantly more effective than when applied with the applicator. It is possible that higher contact pressure was achieved with the forceps during application, because in trials 2, 4, 5, and 6, the efficacy of P/CHG did not differ between applicator and forceps application. This is supported by the reported differences in the skin compatibility in trial 1. The equivalence of antiseptic efficacy using the applicator or the conventional method regarding the reduction of the bacterial load on the skin has been confirmed by McDonald et al. [8].

When interpreting the results, it should be noted that the use of the applicator or forceps under laboratory conditions is not comparable to clinical use, as testing skin antiseptics according to the VAH method [5] ensures standardized application for both techniques, which is not the case under clinical conditions. In the latter, the potential advantages of the applicator are volume control of the antiseptic, time savings, and reduction of dosage errors [7]. The advantage of the applicator was confirmed both in terms of time savings and compliance with critical application steps [9]. In >5,400 interventions, antiseptic application using a 1-step procedure was found to correlate significantly with compliance with the instructions for use (application and drying time). In contrast, 2-step or multi-step procedures correlated significantly with non-compliance with at least one of these steps [10]. In addition, when using the applicator, a significantly more favorable result was observed in terms of unwanted fluid accumulation and better foil adhesion [11]. 

The VAH method requires antiseptic application by back-and-forth friction without applying pressure, instead of concentric circle application when testing skin antiseptics [5]. A randomized open-label study (n=113) confirmed that the lg reduction was significantly higher with the back-and-forth friction method compared to concentric circular application [12]. Using the applicator for 30 s of back-and-forth friction instead of concentric circular application was also superior in terms of wetting [13]. The explanation can be found in the anatomy of the skin, where the back-and-forth friction technique achieves a peeling-like effect in deeper cell layers, focusing on the incision site [11], [14]. In contrast, concentric circles can lead to insufficient penetration of the antiseptic into the cracks and crevices of the epidermis [15]. In a consensus process involving 306 European orthopedic surgeons, 93% agreed that greater importance must be placed on applying antiseptic to the incision site. 90% considered the application method to be highly important, and 97% believed that standardized use of antiseptic improves the prevention of prosthetic infections [16]. In conclusion, various guidelines recommend the use of the single-use applicator with light pressure and repeated back-and-forth movements for 30 seconds, followed by air drying for at least 2 minutes [17], [18], [19], [20]. 

Another finding of the present study was that the reference standard 70% v/v propan-2-ol for testing skin antiseptics in Germany [5] tended to be or was significantly less antiseptically effective than the P/CHG antiseptic. This confirms the clinical study in which the P/CHG antiseptic significantly exceeded the effectiveness of 70% v/v propan-2-ol in terms of reducing SSI. In the multivariable analysis, skin antisepsis with P/CHG was an independent factor for the reduced incidence of SSI [21].

In this context, it is worth mentioning the a study [22] in which skin antisepsis with application of 0.5% CHG in 70% v/v propan-2-ol (n=41) before harvesting the long saphenous vein (used in coronary artery bypass grafting) was compared with 2% CHG in 70% v/v propan-2-ol (n=44) applied with swabs. Although the study did not directly investigate the advantages of the applicator itself, the use of the antiseptic with the higher concentration of CHG resulted in significantly fewer positive test cultures obtained from the skin after 2 minutes, after the incision, and after 24 hours. However, the rate of superficial SSIs was only slightly lower after application of the antiseptic with 2% CHG, whilst the study was not conducted with SSI as a primary end point [22].

The results regarding sustainability are surprising. When comparing the single-use applicator with forceps and swab, the amount of solid waste is more than four times less (111.2 g vs. 572.7 g) and the discarded fluid differs by 200 ml; this is because there is no antiseptic fluid waste when using the applicator [23]. The completion of one preoperative skin preparation procedure with BD ChloraPrep™ 26-mL applicators may result in 49% less CO_2_-eq emissions when compared to the use of bulk antiseptic solutions, resulting in a lower overall carbon footprint [1]. 

## Conclusions

While in trial 4 the antiseptic P/CHG was significantly more effective than P, in trial 5, the P/CHG antiseptic only tendended to be more effective than P. Since the higher efficacy of the P/CHG antiseptic compared to P was clinically confirmed by the criterion SSI rate [21], it can be concluded that the VAH test model method 13 for certification of skin antiseptics in Germany has a good predictive value.

According to the GRADE (Grading of Recommendations Assessment, Development and Evaluation)-based classification of evidence, the results presented here are of “high evidence”. Thus, for preoperative skin antisepsis, P/CHG is to be used instead of alcohol-based formulations lacking CHG, due to the sustained efficacy of CHG. 

Because the antiseptic efficacy of applying the antiseptic with forceps and gauze swab or single-use applicator does not differ on skin with few or many sebaceous glands, the single-use applicator should be preferred, as it not only promotes standardization but is also economically and ecologically more sustainable.

## Notes

### Competing interests

The author, Koburger-Jannsen T, discloses a potential conflict of interest, because the tests were carried out by Hygiene-Nord on behalf of BBraun Sempach, Switzerland, and Ecolab Deutschland GmbH, Monheim, Germany.

The author Kramer A declares that he has no competing interests.

### Ethical approval 

The ethical background is given in the methods section.

### Funding

None. 

### Acknowledgments

The tests were carried out at the Hygiene Nord GmbH test laboratory in Greifswald, which is accredited according to DIN EN ISO/IEC 17025:2018, as part of the application for listing by the VAH.

We would like to thank BBraun Sempach, Switzerland, Becton Dickinson, Switzerland, and Ecolab Deutschland GmbH, Monheim, Germany, for releasing the test results for evaluation in this publication.

### Author’s ORCID

- Kramer A: https://orcid.org/0000-0003-4193-2149

## Figures and Tables

**Table 1:**
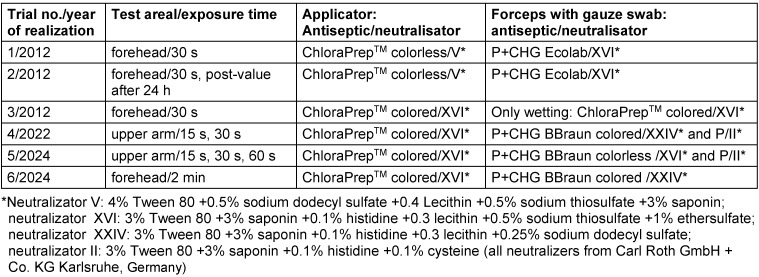
Characteristics of the six trials

**Table 2:**
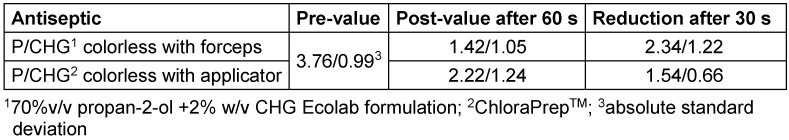
Logarithmic pre-values, post-values, and reduction values after 30 s continuous rubbing on the forehead (n=20)

**Table 3:**
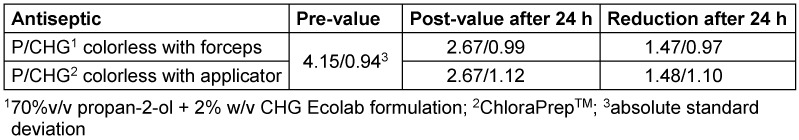
Logarithmic pre-values, post-values and reduction values 24 hours after 30 s continuous rub-in on the forehead (n=20)

**Table 4:**
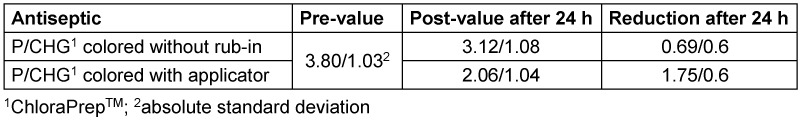
Logarithmic pre-values, post-values and reduction values on the forehead (n=26)

**Table 5:**
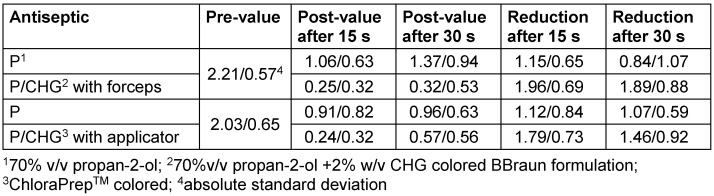
Logarithmic pre-values, post-values, and reduction values after 15 and 30 s on the upper arm (each n=20)

**Table 6:**
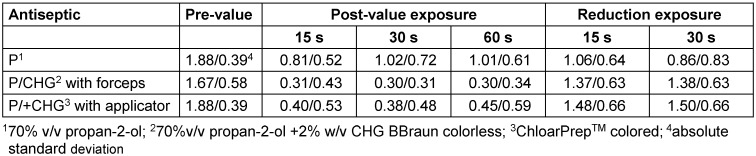
Logarithmic pre-values, post-values and reduction values after 15 s, 30 s and 60 s exposure on the upper arm (each n=23)

**Table 7:**
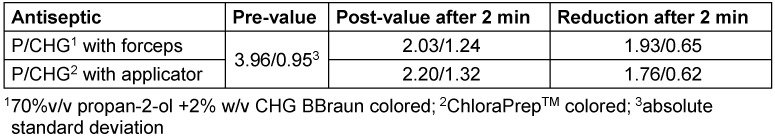
Logarithmic pre-values, post-values, and reduction values after 2 min exposure on the forehead (each n=23)
